# Does the cell number of 0PN embryos on day 3 affect pregnancy and neonatal outcomes following single blastocyst transfer?

**DOI:** 10.1186/s12884-022-04492-7

**Published:** 2022-03-12

**Authors:** Chen Chen, Wenzhi Li, Mingru Yin, Menghui Li, Ling Wu, Jiqiang Si, Leiwen Zhao, Bin Li, Zheng Yan, Qifeng Lyu

**Affiliations:** Department of Assisted Reproduction, Shanghai Ninth People’s Hospital, Shanghai Jiao Tong University School of Medicine, 639 Zhizaoju Rd, Shanghai, 200011 China

**Keywords:** 0PN, Cell number, Blastocyst culture, Pregnancy outcomes, Neonatal outcomes

## Abstract

**Background:**

0PN zygotes have a low cleavage rate, and the clinical outcomes of cleavage-stage embryo transfers are unsatisfactory. Blastocyst culturing is used to screen 0PN embryos, but whether the cell number of 0PN embryos on day 3 affects the clinical outcomes following single blastocyst transfer is unknown and would be helpful in evaluating the clinical value of these embryos.

**Methods:**

This retrospective study compared 46,804 0PN zygotes, 242 0PN frozen-thawed single blastocyst transfers, and 92 corresponding 0PN singletons with 232,441 2PN zygotes, 3563 2PN frozen-thawed single blastocyst transfers, and 1250 2PN singletons from January 2015 to October 2019 at a tertiary-care academic medical centre. The 0PN and 2PN embryos were divided into two groups: the group with < 6 cells on day 3 and that with ≥ 6 cells. Embryo development, subsequent pregnancy and neonatal outcomes were compared between the two groups.

**Results:**

The cleavage and available blastocyst rates of the 0PN zygotes were much lower than those of the 2PN zygotes (25.9% vs. 97.4%, *P* < 0.001; 13.9% vs. 23.4%, *P* < 0.001). In the < 6 cells group, the available blastocyst rate of the cleaved 0PN embryos was significantly lower than that of the 2PN embryos (2.5% vs. 12.7%, *P* < 0.001). However, in the ≥ 6 cells group, the available blastocyst rate of the 0PN cleaved embryos significantly improved, although it was slightly lower than that of the 2PN embryos (33.9% vs. 35.7%, *P* = 0.014). Importantly, compared to those of the 2PN single blastocyst transfers, the clinical pregnancy rate, live birth rate, Z-score and malformation rate of the 0PN single blastocyst transfers were not significantly different in either the < 6 cells group (30.4% vs. 39.8%, *P* = 0.362; 30.4% vs. 31.3%, *P* = 0.932; 0.89 ± 0.90 vs. 0.42 ± 1.02, *P* = 0.161; 0% vs. 2.6%, *P* = 1.000) or the ≥ 6 cells group (50.7% vs. 46.6%, *P* = 0.246; 39.7% vs. 38.3%, *P* = 0.677; 0.50 ± 1.23 vs. 0.47 ± 1.11, *P* = 0.861; 2.4% vs. 1.8%, *P* = 1.000).

**Conclusions:**

The cell number on day 3 of 0PN embryos affected the subsequent formation of blastocysts but did not influence the subsequent pregnancy and neonatal outcomes of 0PN single blastocyst transfers, which may be beneficial to clinicians counselling patients on the clinical value of 0PN embryos.

**Supplementary Information:**

The online version contains supplementary material available at 10.1186/s12884-022-04492-7.

## Background

In assisted reproductive technology (ART), the presence of two pronuclei (2PN) with two polar bodies is a symbol of normal fertilization, while nonpronuclear (0PN) zygotes are typically the result of abnormal or failed fertilization. Generally, embryos derived from 0PN zygotes are not recommended for clinical use [1]. However, several studies have reported that these seemingly abnormal embryos can result in successful pregnancies and live birth after transfer, which has raised the value of 0PN embryos, especially for patients without two pronuclei (2PN) embryos [2–9]. Consequently, revealing the clinical value of 0PN embryos is important. This not only saves precious clinical resources but also increases the chance of pregnancy for female patients, especially older patients with reduced ovarian reserve.

Previous studies have reported that cleavage-stage 0PN embryos have significantly worse clinical outcomes than cleavage-stage 2PN embryos [3, 10]. However, Li et al. (2015) reported that 0PN blastocysts had similar clinical outcomes to 2PN blastocysts and advised culturing 0PN embryos to the blastocyst stage for selection. Consistent with these findings, recent studies have shown that blastocyst culture of 0PN embryos can result in notable clinical pregnancies and live births [5, 7–9]. The quality and implantation potential of blastocysts can be predicted by the morphological parameters of cleavage-stage embryos, including the day 3 blastomere number, fragmentation and symmetry [11, 12]. However, the value of 0PN blastocysts originating from cleaved embryos with different cell numbers on day 3 is still unclear. A recent study reported that a low cell number (< 6 cells) was independently associated with a decreased live birth rate in single blastocyst transfers [13]. Thus, whether 0PN blastocysts originating from day 3 embryos with different cell numbers display different clinical outcomes needs further study. In addition, the health risks of 0PN offspring have gained extensive attention. To date, one retrospective study has reported that 0PN singletons had higher birthweights and Z-scores than 2PN singletons and that a greater proportion of 0PN singletons were very large for gestational age [8]; other previous studies, however, lacked information on neonatal outcomes from 0PN embryo transfers [3, 5, 7, 9, 14]. Thus, the safety of 0PN embryo transfers needs to be further investigated [8].

To address these issues, we divided 0PN embryos according to day 3 cell number (< 6 cells and ≥ 6 cells), analysed their embryonic development results, and retrospectively compared the pregnancy and neonatal outcomes of subsequent single 0PN blastocyst transfers with single 2PN blastocyst transfers. In summary, this study provides information about the clinical value of 0PN embryos and the safety of 0PN offspring originating from different cell numbers on day 3.

## Methods

### Study design and patients

This study retrospectively analysed the embryonic outcomes of 0PN zygotes observed in oocyte retrieval cycles and the clinical outcomes of the subsequent single 0PN blastocyst transfer cycles from January 2015 to October 2019 in a tertiary-care academic medical centre. The control group was 2PN embryos, and the embryo development and clinical outcomes were compared between the 0PN and 2PN groups. The exclusion criteria were as follows: in vitro maturation cycles; loss to follow-up; vanishing twin syndrome; and multifoetal pregnancy reduction. None of the cycles included in the study used preimplantation genetic testing (PGT) since PGT is not performed at this centre. Finally, a total of 51,739 oocyte retrieval cycles and corresponding 3805 single blastocyst transfer cycles were included. The details of the ART treatment were recorded in the electronic medical database and were consistent with the requirements of the Technical Standard for Human-Assisted Reproduction issued by the Chinese Ministry of Health. The study was approved by the institutional review board of Shanghai Ninth People's Hospital affiliated with Shanghai Jiao Tong University School of Medicine, and no informed consent was required because the study was a retrospective study.

### Laboratory protocols

Approximately 34–36 h after trigger, the oocytes were retrieved and fertilized with the use of in vitro fertilization (IVF) or intracytoplasmic sperm injection (ICSI) as described in our previous study [15]. Briefly, in IVF, spermatozoa were collected by density gradient centrifugation and swim-up methods, and the oocytes were inseminated with 50,000 motile sperm cells/mL in the insemination dish. In ICSI, the removal of cumulus cells was performed after 2–3 h of oocyte retrieval, and the injection was carefully performed within 2 h of oocyte denudation. Approximately 16–18 h after insemination, fertilization was assessed by the presence of pronuclei. Throughout the entire developmental stage, the embryos were cultured in Continuous Single Culture (CSC, Irvine Scientific, USA) and incubated with a humidified atmosphere containing 5% O_2_ and 6% CO_2_ at 37 °C. On day 3, the cell number and morphological grade of the embryos were recorded based on the ASEBIR embryo assessment criteria [16]. In general, 2PN good-quality embryos (Grades I and II) were selected for transfer or vitrification, while poor-quality embryos (Grades III and IV) were subjected to extended culture in clinical practice. During the period of our study, the strategy of our centre was to subject 0PN embryos to extended blastocyst culture. Blastocysts were scored in accordance with the Gardener and Schoolcraft scoring system [17], and Grade ≥ 3BB was defined as available in this study. The procedures of vitrification and thawing for blastocysts were described in our previous study [18].

### Transfer cycles and outcome parameters

The endometrium was prepared by a modified natural cycle, stimulated cycle or artificial cycle according to the individual conditions [19]. In the current study, only one available blastocyst was transferred in every transfer cycle. After embryo transfers, luteal support was initiated as described previously [19].

The serum human chorionic gonadotropin (hCG) concentration of the patients was measured on the 12th day after blastocyst transfer. Biochemical pregnancy was defined as a positive hCG test. Clinical pregnancy was confirmed by the observation of a gestational sac on ultrasound examination on the 35th day after embryo transfer. Miscarriage was defined as intrauterine pregnancy loss before the 24th gestational week. Ectopic pregnancy was defined as a pregnancy that occurred outside of the uterine cavity. Live birth was defined as delivery of a living baby at ≥ 24 weeks gestation. Gestational age was calculated by adding 19 days from the date of blastocyst transfer. Very low birthweight (VLBW), low birthweight (LBW), and high birthweight (HBW) were defined as birthweights < 1500 g, < 2500 g, and > 4500 g, respectively. Z-scores and birthweight percentiles were calculated on the basis of the INTERGROWTH-21st reference adjusted by neonatal sex and gestational age [20]. Very small for gestational age (VSGA), small for gestational age (SGA), large for gestational age (LGA) and very large for gestational age (VLGA) were defined as birthweights in the < 3rd, < 10th, > 90th and > 97th percentiles, respectively. Congenital malformations were confirmed, diagnosed and coded based on the International Classification of Diseases, 10th Revision (ICD-10).

### Statistical analysis

Statistical analyses were performed with the Statistical Package for the Social Sciences version 25.0 software (SPSS Inc., Chicago, USA). A significant difference was considered at *P* < 0.05, and all *P* values were based on two-sided tests. The normality of the quantitative variables was tested by the Kolmogorov–Smirnova and Shapiro–Wilk tests, the graphical illustration of histograms and Q-Q plots. The data are presented as the mean ± standard deviation (SD) or the median (first quartile, third quartile) as appropriate. Comparisons of between-group differences were performed with Student’s t test or the Mann–Whitney U test. For qualitative variables, the chi-square test or Fisher’s exact test was applied to analyse the differences, and the data are presented as % (n/N).

## Results

### Development and utility of 0PN embryos

**Fig. 1:**
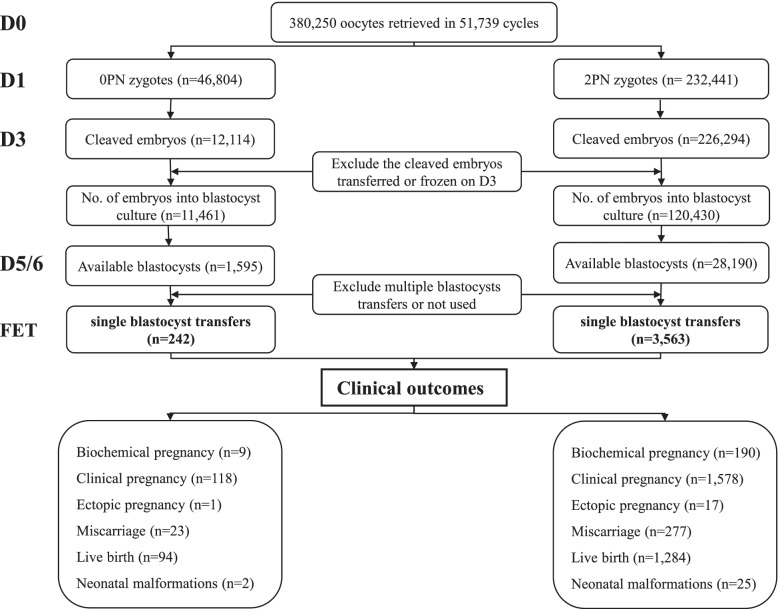
The flow chart of the study. 0PN, nonpronuclear; 2PN, two pronuclei; FET, frozen-thawed embryo transfer; D0, day 0; D1, day 1; D3, day 3; D5/6, day 5/6

The flow chart of the study is shown in Fig. 1. A total of 51,739 oocyte retrieval cycles were completed, and 380,250 oocytes were obtained between January 2015 and October 2019. A total of 46,804 0PN zygotes and 232,441 2PN zygotes were observed. The cleavage and available blastocyst rates of the 0PN zygotes were much lower than those of the 2PN zygotes (25.9% vs. 97.4%, *P* < 0.001; 13.9% vs. 23.4%, *P* < 0.001; Table 1). We noticed that the cleaved 0PN embryos had a higher proportion of < 6 cells than ≥ 6 cells (< 6 cells vs. ≥ 6 cells: 61.5% vs. 38.5%), but the 2PN embryos showed the opposite trend (29.7% vs. 70.3%, Table 1). Then, we divided the cleaved embryos into two groups according to the cell number on day 3 (< 6 cells and ≥ 6 cells) and found that the available blastocyst rate of the cleaved 0PN embryos was significantly lower than that of the 2PN embryos in the < 6 cells group (2.5% vs. 12.7%, *P* < 0.001, Table 1). In addition, in the ≥ 6 cells group, the blastocyst culture of 0PN day 3 embryos obtained a very close rate of available blastocysts compared with the 2PN embryos (33.9% vs. 35.7%), but there was still a significant difference (*P* = 0.014, Table 1). In addition, a higher proportion of 0PN blastocysts on day 5 than 2PN blastocysts was observed in the ≥ 6 cells group (43.5% vs. 27.0%, *P* < 0.001, Table 1). Finally, 1595 0PN and 28,190 2PN available blastocysts were vitrified.

**Table 1:** Development and utility of 0PN embryos divided by cell number on day 3

	**0PN**	**2PN**	***P*-Value**
**No. of zygotes (n)**	46,804	232,441	/
**Cleavage rate (%)**	25.9 (12,114/46804)	97.4 (226,294/232441)	< 0.001
< 6 cells (%)	61.5 (7450/12114)	29.7 (67,261/226294)	
≥ 6 cells (%)	38.5 (4664/12114)	70.3 (159,033/226294)	
**No. of embryos into blastocyst culture (n)**	11,461	120,430	/
**Available blastocyst rate, ABR (%)**	13.9 (1595/11461)	23.4 (28,190/120,430)	< 0.001
< 6 cells (%)	2.5 (182/7287)	12.7 (8145/64359)	< 0.001
Day 5 (%)	14.3 (26/182)	12.6 (1023/8145)	0.488
Day 6 (%)	85.7 (156/182)	87.4 (7122/8145)	
≥ 6 cells (%)	33.9 (1413/4174)	35.7 (20,045/56071)	0.014
Day 5 (%)	43.5 (615/1413)	27.0 (5409/20045)	< 0.001
Day 6 (%)	56.5 (798/1413)	73.0 (14,636/20045)	

*0PN* Nonpronuclear, *2PN* Two pronuclei

### Pregnancy outcomes of single 0PN blastocyst transfers

A total of 242 single 0PN blastocyst transfers and 3563 single 2PN blastocyst transfers were performed during the study period (Fig. 1). Considering that the transferred blastocysts originated from cleaved embryos with different cell numbers on day 3, we divided the cycles into the < 6 cells group and ≥ 6 cells group to compare the pregnancy outcomes. The baseline demographic and transfer cycle characteristics were similar between the groups, as shown in Additional file 1.

In the < 6 cells group, the blastocyst development day composition was similar between the two groups (Table 2). The clinical pregnancy, ectopic pregnancy, miscarriage and live birth rates of the 0PN cycles were similar to those of the 2PN cycles (30.4% vs. 39.8%, *P* = 0.362; 0% vs. 0.9%, *P* = 1.000; 0% vs. 20.6%, *P* = 0.383; 30.4% vs. 31.3%, *P* = 0.932; Table 2).

**Table 2:** Pregnancy outcomes of single 0PN blastocyst transfers in two groups

	** < 6 cells**		***P*-Value**	** ≥ 6 cells**		***P*-Value**
	**0PN (*N* = 23)**	**2PN (*N* = 1145)**		**0PN (*N* = 219)**	**2PN (*N*** **= 2418)**	
**Embryo development stage**			0.993			< 0.001
Day 5	17.4 (4/23)	15.1 (173/1145)		56.2 (123/219)	35.6 (860/2418)	
Day 6	82.6 (19/23)	84.9 (972/1145)		43.8 (96/219)	64.4 (1558/2418)	
**Biochemical pregnancy rate (%)**	0 (0/23)	5.6 (64/1145)	0.482	4.1 (9/219)	5.2 (126/2418)	0.479
**Clinical pregnancy rate (%)**	30.4 (7/23)	39.8 (456/1145)	0.362	50.7 (111/219)	46.6 (1122/2418)	0.246
**Ectopic pregnancy rate (%)**	0 (0/7)	0.9 (4/456)	1.000	0.9 (1/111)	1.2 (13/1114)	1.000
**Miscarriage rate (%)**	0 (0/7)	20.6 (94/456)	0.383	20.7 (23/111)	16.7 (183/1114)	0.282
**Live birth rate (%)**	30.4 (7/23)	31.3 (358/1145)	0.932	39.7 (87/219)	38.3 (926/2418)	0.677
Singleton	30.4 (7/23)	30.4 (348/1145)		38.8 (85/219)	37.3 (902/2418)	
Multiple	0 (0/23)	0.9 (10/1145)		0.9 (2/219)	1.0 (24/2418)	

*0PN* Nonpronuclear, *2PN* Two pronuclei

In the ≥ 6 cells group, the clinical pregnancy, ectopic pregnancy, miscarriage and live birth rates also showed no significant difference between the 0PN and 2PN groups (50.7% vs. 46.6%, *P* = 0.246; 0.9% vs. 1.2%, *P* = 1.000; 20.7% vs. 16.7%, *P* = 0.282; 39.7% vs. 38.3%, *P* = 0.677; Table 2). However, the composition of blastocyst development stage in the 0PN group was different from that in the 2PN group (day 5: 56.2% vs. 35.6%, *P* < 0.001; day 6: 43.8% vs. 64.4%, *P* < 0.001; Table 2).

Subsequently, the 0PN blastocysts were subdivided into seven groups (≤ 4 cells, 5 cells, 6 cells, 7 cells, 8 cells, 9 cells and ≥ 10 cells) based on the cell number of the 0PN day 3 cleavage embryos to further analyse the effects on clinical pregnancy and live birth rates. The results showed that no significant differences were found among any of the subgroups between the 0PN and 2PN blastocysts (Table 3).

**Table 3:** Clinical pregnancy and live birth rates of single 0PN blastocyst transfers divided by cell number on day 3

	**Clinical pregnancy rate (%)**			**Live birth rate (%)**		
	**0PN**	**2PN**	***P*-Value**	**0PN**	**2PN**	***P*-Value**
**Subgroups**						
≤ 4 cells	26.7% (4/15)	39.2% (185/472)	0.327	26.7% (4/15)	29.0% (137/472)	1.000
5 cells	37.5% (3/8)	40.3% (271/673)	1.000	37.5% (3/8)	32.8% (221/673)	1.000
6 cells	58.6% (17/29)	43.3% (367/848)	0.102	37.9% (11/29)	35.7% (303/848)	0.808
7 cells	52.6% (20/38)	48.1% (361/751)	0.583	42.1% (16/38)	39.3% (295/751)	0.728
8 cells	47.9% (34/71)	47.5% (289/608)	0.955	43.7% (31/71)	38.7% (235/608)	0.413
9 cells	50.0% (11/22)	47.7% (41/86)	0.846	36.4% (8/22)	44.2% (38/86)	0.508
≥ 10 cells	49.2% (29/59)	51.2% (64/125)	0.795	35.6% (21/59)	44.0% (55/125)	0.280

*0PN* Nonpronuclear, *2PN* Two pronuclei

### Neonatal outcomes of singleton live birth after single 0PN blastocyst transfers

In the < 6 cells group, 7 0PN singletons and 348 2PN singletons were delivered. The mean gestational age, birthweight, Z-score and malformation rate were similar between the two groups (38.1 ± 1.8 vs. 38.2 ± 1.8, *P* = 0.954; 3457.9 ± 411.6 vs. 3313.5 ± 504.7, *P* = 0.517; 0.89 ± 0.90 vs. 0.42 ± 1.02, *P* = 0.161; 0% vs. 2.6%, *P* = 1.000; Table 4). In the ≥ 6 cells group, 85 0PN singletons and 891 2PN singletons were delivered. The mean gestational age, birthweight, Z-score and malformation rate were also similar between the two groups (37.9 ± 2.2 vs. 38.2 ± 1.8, *P* = 0.115; 3324.2 ± 550.4 vs. 3276.0 ± 598.8, *P* = 0.397; 0.50 ± 1.23 vs. 0.47 ± 1.11, *P* = 0.861; 2.4% vs. 1.8%, *P* = 1.000; Table 4). In addition, no significant differences were observed in the rates of VLBW, LBW, HBW, VSGA, SGA, LGA and VLGA between the 0PN and 2PN singletons in either the < 6 cells or ≥ 6 cells group.

**Table 4:** Neonatal outcomes of 0PN singletons after single blastocyst transfers in two groups

	** < 6 cells**		***P*-Value**	** ≥ 6 cells**		***P*-Value**
	**0PN (*N*** **= 7)**	**2PN (*N*** = 348)		**0PN (*N* = 85)**	**2PN (*N* = 902)**	
**Mean gestational age (weeks)**	38.1 ± 1.8	38.2 ± 1.8	0.954	37.9 ± 2.2	38.2 ± 1.8	0.115
**Birthweight (g)**	3,457.9 ± 411.6	3,313.5 ± 504.7	0.517	3,324.2 ± 550.4	3,276.0 ± 598.8	0.397
VLBW (< 1500 g)	0 (0/7)	1.1 (4/348)	1.000	1.2 (1/85)	1.2 (11/902)	1.000
LBW (< 2500 g)	0 (0/7)	3.4 (12/348)	1.000	7.1 (6/85)	5.2 (47/902)	0.638
HBW (> 4500 g)	0 (0/7)	0.6 (2/348)	1.000	1.2 (1/85)	1.0 (9/902)	1.000
**Z-score**	0.89 ± 0.90	0.42 ± 1.02	0.161	0.50 ± 1.23	0.47 ± 1.11	0.861
**VSGA rate (%)**	0 (0/7)	0.9 (3/348)	1.000	3.5 (3/85)	1.8 (16/902)	0.476
**SGA rate (%)**	0 (0/7)	3.7 (13/348)	1.000	5.9 (5/85)	4.2 (38/902)	0.658
**LGA rate (%)**	42.9 (3/7)	19.8 (69/348)	0.305	21.2 (18/85)	22.2 (200/902)	0.832
**VLGA rate (%)**	0 (0/7)	9.5 (33/348)	0.843	12.9 (11/85)	9.9 (89/902)	0.369
**Malformation rate (%)**	0 (0/7)	2.6 (9/348)	1.000	2.4 (2/85)	1.8 (16/902)	1.000

*0PN* Nonpronuclear, *2PN* Two pronuclei

## Discussion

It is critical to understand the clinical value and safety of 0PN embryos in ART treatment. Our study showed that in the < 6 cells group, 0PN embryos had a significantly lower available blastocyst rate than 2PN embryos, but after 0PN embryos were cultured to the blastocyst stage, they showed pregnancy outcomes comparable to those of the 2PN group in single blastocyst transfer cycles. In the ≥ 6 cells group, the available blastocyst rates were almost the same between the 0PN and 2PN embryos, and the rates of clinical pregnancy and live birth were similar between the 0PN and 2PN blastocyst transfers. Importantly, no differences were observed in the neonatal outcomes between the 0PN and 2PN singletons. These data suggested that if 2PN day 3 embryos are not available or are limited in number, 0PN day 3 embryos of ≥ 6 cells should be preferred for blastocyst culture, and 0PN blastocysts can also be transferred without increased birth defect risk.

Previous studies have reported that the appearance of 0PN zygotes is common upon fertilization and that the majority of them may originate from failed fertilization and cannot be cleaved afterwards [3, 5, 21]. Consistent with previous studies, our study also observed that the cleavage rate of 0PN zygotes was significantly lower than that of 2PN zygotes, indicating that 0PN zygotes had a higher possibility of fertilization failure. Then, we grouped 0PN and 2PN embryos into < 6 cells and ≥ 6 cells groups to analyse subsequent embryonic developmental competence. In the < 6 cells group, the 0PN embryos had a much lower available blastocyst rate than the 2PN embryos. Moreover, we found that cleaved 0PN embryos had a higher proportion of < 6 cells on day 3 than 2PN embryos. Previous studies reported that the cleavage of 0PN zygotes was partly caused by parthenogenetic activation or abnormal fertilization [3, 21]. We supposed that some of the 0PN embryos in the < 6 cells group originated from parthenogenetic activation or abnormal fertilization and could not develop into a blastocyst. In the ≥ 6 cells group, our data showed a similar available blastocyst rate between 0 and 2PN embryos. We also found a higher proportion of 0PN blastocysts on day 5 than 2PN blastocysts in the ≥ 6 cells group. A previous study reported that embryo morphology on day 3 influenced the speed of blastocyst formation [11, 12, 22], and the existing literature showed evidence supporting that day 5 blastocysts are associated with a higher live birth rate than day 6 blastocysts in the frozen-thawed cycle [23, 24]. The difference in blastocyst proportion between day 5 and day 6 may be because of the different selection and culture strategies between 0 and 2PN embryos on day 3 at our centre. Generally, only poor-quality 2PN embryos are subjected to extended culture; however, all 0PN embryos receive extended blastocyst culture at our centre.

Our study also showed that the pregnancy outcomes of 0PN blastocyst transfers were comparable with those of 2PN blastocyst transfers in both the < 6- and ≥ 6 cells groups, suggesting that blastocyst culture was of great benefit for selecting normal 0PN embryos for transfer [3, 8]. In blastocyst culture, the embryo genome is activated, and many embryos with abnormal chromosomes are eliminated [25–27]. Two recent studies reported that compared to 2PN blastocysts, 0PN blastocysts exhibit similar rates of normal chromosomal status (64.71% vs. 69.39%) and biparental diploid status (75.51% vs. 80.13%) [6, 28]. Commonly, the blastomere number reflects the embryo developmental speed and is used to assess embryo quality [12, 29]. In this study, although the available blastocyst rate of 0PN embryos was significantly lower than that of 2PN embryos in the < 6 cells group, the live birth rate was not impaired, and the biochemical pregnancy, ectopic pregnancy, and miscarriage rates were not increased. This finding provides meaningful information about the safety and clinical value of 0PN blastocysts derived from < 6 cells embryos on day 3.

Generally, fertilization and pronuclear formation are checked by embryologists within 16–18 h after insemination [30]. Sometimes pronuclei are not visible at this time since pronuclear membrane breakdown could occur as early as 6.16 h, and pronuclei might appear as late as 29.4 h after microinjection [31, 32]. Some studies indicate that a certain number of embryos are wrongly divided into an abnormal fertilized group under an optical microscope, while they had a normal diploid status sin a genetic test [33, 34]. Thus, 0PN embryos that can develop into blastocysts may be derived from zygotes with normal fertilization but with accelerated breakdown of the pronuclear membrane [3–5, 21]. The use of time lapse (TL) technology may be a better choice for pronuclear checks. It could provide accurate observations and more details about dynamic changes in pronuclei and help in identifying the origin of 0PN zygotes [35, 36].

For neonatal outcomes, a recent study showed that compared with 2PN singletons, 0PN singletons had an increased birthweight and Z-score and a higher risk of VLGA, which was not observed in our study. One possible explanation was that patient characteristics, such as maternal weight gain during pregnancy, parity, subfertility diagnosis and ovarian response, were not exactly the same among the studies but may influence the newborns’ birthweight [37–39]. Most importantly, the malformation risk, an important issue in ART, was not increased by 0PN blastocyst transfers in either our study or Li’s study [40].

In addition, the embryo quality and chances of achieving pregnancy were also affected by the patient’s particular conditions, such as endometriosis, polycystic ovary syndrome (PCOS) and endometriotic ovarian cysts [41–44]. Thus, various modified and personalized ovarian stimulation strategies were adopted by infertile patients to improve the outcomes. Previous studies reported that the IVF outcomes of patients with endometriosis were improved by pretreatment with DNG, and myo-inositol is of proven utility in those with PCOS [45, 46]. Our centre also published some work in this field on the basis of a novel ovarian stimulation strategy named progestin primed ovarian stimulation (PPOS) [15, 47, 48].

The limitations of our study are its retrospective design, the small number of 0PN singletons, and the lack of other screening methods, such as PGT. PGT is recommended to exclude nondiploid embryos for transfers, which could reduce the chance of miscarriage [49, 50]. The inclusion of developmentally competent 0PN and 1PN embryos after genome-wide haplotyping allows embryo transfers to reach 81% and increases the live birth rate by 75% in PGT cycles [6]. Thus, a larger study that considers PGT and includes strict long-term follow-up of 0PN offspring is necessary to confirm our findings and validate the use of 0PN blastocyst transfer.

## Conclusions

This study assessed the clinical value of 0PN blastocysts based on cell number on day 3. The developmental competence of 0PN embryos was different from that of 2PN embryos, but the pregnancy and neonatal outcomes of 0PN blastocyst transfers were similar to those of 2PN blastocyst transfers. Our study highlights the appreciable clinical value of 0PN blastocysts derived from < 6 cells embryos on day 3. Compared with 2PN embryos, < 6 cells 0PN embryos showed a significantly lower available blastocyst rate but obtained similar pregnancy and neonatal outcomes when they reached the blastocyst stage. In conclusion, our results provide evidence of the effectiveness of blastocyst culture of 0PN zygotes and the value and safety of 0PN blastocyst transfers, especially regarding the live birth rate and malformation risks of 0PN singletons.

## Supplementary Information

**Additional file 1.** Main characteristics of patients and treatment of single 0PN and 2PN blastocyst transfers divided by cell number on day 3.

## Data Availability

The data is not publicly available and please contact corresponding author for data requests.

## Abbreviations

2PN: Presence of two pronuclei
0PN: Nonpronuclear
ART: Assisted reproductive technology
HBW: High birthweight
ICSI: Intracytoplasmic sperm injection
IVF: In vitro fertilization
LBW: Low birthweight
LGA: Large for gestational age
PGT: Preimplantation genetic testing
SGA: Small for gestational age
TL: Time lapse
VLBW: Very low birthweight
VLGA: Very large for gestational age
VSGA: Very small for gestational age
