# Supplementary material for: Does the cell number of 0PN embryos on day 3 affect pregnancy and neonatal outcomes following single blastocyst transfer?
Source: BMC Pregnancy Childbirth. 2022 Mar 12;22:200. doi: 10.1186/s12884-022-04492-7 (PMC8918324; doi:10.1186/s12884-022-04492-7)
Supplement: Supplementary file 1 — Additional file 1. Main characteristics of patients and treatment of single 0PN and 2PN blastocyst transfers divided by cell number on day 3. [file 12884_2022_4492_MOESM1_ESM.docx]

**Additional file 1.** **Main characteristics of patients and treatment of single 0PN and 2PN blastocyst transfers divided by cell number on day 3.**

|  | **< 6 cells** | | ***P*-Value** | **≥ 6 cells** | | ***P*-Value** |
| --- | --- | --- | --- | --- | --- | --- |
|  | **0PN (N = 23)** | **2PN (N = 1145)** |  | **0PN (N = 219)** | **2PN (N = 2418)** |  |
| **Maternal age (years)** | 33.0 ± 4.7 | 33.8 ± 4.6 | 0.575 | 33.4 ± 5.5 | 33.3 ± 4.6 | 0.399 |
| **Maternal BMI (kg/m2)** | 21.8 ± 2.8 | 21.6 ± 2.7 | 0.386 | 22.1 ± 2.8 | 21.9 ± 2.6 | 0.312 |
| **Type of infertility (%)** |  |  | 0.286 |  |  | 0.611 |
| Primary | 43.5 (10/23) | 54.7 (626/1145) |  | 52.5 (115/219) | 54.3 (1313/2418) |  |
| Secondary | 56.5 (13/23) | 45.3 (519/1145) |  | 47.5 (104/219) | 45.7 (1105/2418) |  |
| **Infertility duration (years)** | 3.5 ± 3.3 | 3.4 ± 3.0 | 0.620 | 3.2 ± 2.9 | 3.1 ± 2.9 | 0.828 |
| **Gravidity** | 0 (0,1) | 0 (0,1) | 0.228 | 0 (0,1) | 0 (0,1) | 0.362 |
| **Parity** | 0 (0,0) | 0 (0,0) | 0.180 | 0 (0,0) | 0 (0,0) | 0.792 |
| **Basal endocrine profiles** |  |  |  |  |  |  |
| FSH, IU/L | 5.2 ± 2.1 | 5.9 ± 1.7 | 0.573 | 5.7 ± 1.6 | 5.4 ± 1.6 | 0.582 |
| LH, IU/L | 4.1 ± 3.1 | 4.0 ± 3.6 | 0.903 | 4.1 ± 3.1 | 4.1 ± 3.4 | 0.357 |
| E2, pg/mL | 46.8 ± 29.5 | 47.5 ± 31.8 | 0.601 | 45.8 ± 31.0 | 46.7 ± 32.4 | 0.219 |
| P, ng/mL | 0.3 ± 0.3 | 0.3 ± 0.2 | 0.817 | 0.2 ± 0.3 | 0.3 ± 0.2 | 0.270 |
| **Endometriosis (%)** | 8.7 (2/23) | 10.0 (115/1145) | 1.000 | 10.5 (23/219) | 8.4 (202/2418) | 0.276 |
| **Adenomyosis (%)** | 4.3 (1/23) | 6.3 (72/1145) | 1.000 | 4.1 (9/219) | 5.0 (120/2418) | 0.575 |
| **Tubal factor infertility (%)** | 82.6 (19/23) | 81.2 (930/1145) | 0.866 | 83.1 (182/219) | 80.9 (1955/2418) | 0.415 |
| **Male factor infertility (%)** | 4.3 (1/23) | 12.8 (146/1145) | 0.229 | 13.2 (29/219) | 12.6 (305/2418) | 0.575 |
| **Endometrial thickness on transfer day (mm)** | 11.6 ± 2.0 | 11.5 ± 2.4 | 0.818 | 11.6 ± 2.2 | 11.4 ± 2.4 | 0.330 |
| **Endometrial preparation (%)** |  |  | 0.813 |  |  | 0.138 |
| Natural cycle | 21.7 (5/23) | 19.4 (222/1145) |  | 12.3 (27/219) | 17.2 (416/2418) |  |
| Mild stimulation | 47.8 (11/23) | 43.8 (501/1145) |  | 51.1 (112/219) | 46.1 (1114/2418) |  |
| Hormone replacement therapy | 30.4 (7/23) | 36.9 (422/1145) |  | 36.5 (80/219) | 36.7 (888/2418) |  |

0PN, nonpronuclear; 2PN, two pronuclei; BMI, body mass index; FSH, follicle-stimulating hormone; LH, luteinizing hormone; E2, estradiol; P, progesterone.
